# Genome-wide distribution of 5-formylcytosine in embryonic stem cells is associated with transcription and depends on thymine DNA glycosylase

**DOI:** 10.1186/gb-2012-13-8-r69

**Published:** 2012-08-17

**Authors:** Eun-Ang Raiber, Dario Beraldi, Gabriella Ficz, Heather E Burgess, Miguel R Branco, Pierre Murat, David Oxley, Michael J Booth, Wolf Reik, Shankar Balasubramanian

**Affiliations:** 1 Department of Chemistry, University of Cambridge, Lensfield Road, Cambridge, CB2 1EW, UK; 2 Cancer Research UK, Cambridge Research Institute, Li Ka Shing Centre, Robinson way, Cambridge, CB2 0RE, UK; 3 Epigenetics Programme, The Babraham Institute, Babraham Research Campus, Cambridge CB22 3AT, UK; 4 Centre for Trophoblast Research, University of Cambridge, Physiology Building, Downing Street, Cambridge CB2 3EG, UK; 5 Proteomics Research Group, The Babraham Institute, Babraham Research Campus, Cambridge CB22 3AT, UK; 6 School of Clinical Medicine, The University of Cambridge, Addenbrooke's Hospital, Hills Road, Cambridge, CB2 0SP, UK

## Abstract

**Background:**

Methylation of cytosine in DNA (5mC) is an important epigenetic mark that is involved in the regulation of genome function. During early embryonic development in mammals, the methylation landscape is dynamically reprogrammed in part through active demethylation. Recent advances have identified key players involved in active demethylation pathways, including oxidation of 5mC to 5-hydroxymethylcytosine (5hmC) and 5-formylcytosine (5fC) by the TET enzymes, and excision of 5fC by the base excision repair enzyme thymine DNA glycosylase (TDG). Here, we provide the first genome-wide map of 5fC in mouse embryonic stem (ES) cells and evaluate potential roles for 5fC in differentiation.

**Results:**

Our method exploits the unique reactivity of 5fC for pulldown and high-throughput sequencing. Genome-wide mapping revealed 5fC enrichment in CpG islands (CGIs) of promoters and exons. CGI promoters in which 5fC was relatively more enriched than 5mC or 5hmC corresponded to transcriptionally active genes. Accordingly, 5fC-rich promoters had elevated H3K4me3 levels, associated with active transcription, and were frequently bound by RNA polymerase II. TDG down-regulation led to 5fC accumulation in CGIs in ES cells, which correlates with increased methylation in these genomic regions during differentiation of ES cells in wild-type and TDG knockout contexts.

**Conclusions:**

Collectively, our data suggest that 5fC plays a role in epigenetic reprogramming within specific genomic regions, which is controlled in part by TDG-mediated excision. Notably, 5fC excision in ES cells is necessary for the correct establishment of CGI methylation patterns during differentiation and hence for appropriate patterns of gene expression during development.

## Background

In mammalian genomes, 5mC plays essential roles in maintaining cellular function and genomic stability, including processes such as × chromosome inactivation, genomic imprinting and transposon silencing [1,2]. During early mammalian development, cytosine methylation undergoes dramatic global changes. Whereas the formation of 5mC marks is well understood, the mechanism of DNA demethylation still remains elusive. Removal of methylation marks can proceed via a passive, replication-dependent pathway. Recent discoveries of other cytosine modifications suggest one possibility of an active demethylation mechanism involving the iterative oxidation of 5-methylcytosine (5mC) to 5-hydroxymethylcytosine (5hmC), 5-formylcytosine (5fC) and 5-carboxycytosine (5caC) by the ten-eleven translocation (TET) family of enzymes, followed by base excision repair by thymine DNA glycosylase (TDG) [3,4]. 5fC has been detected in mouse embryonic stem (ES) cells and brain cortex by thin layer chromatography and tandem liquid chromatography-mass spectrometry. Quantification of 5fC in genomic ES cell DNA showed this modified base to be present at around a level of 0.02 to 0.002% of all cytosine species, which is roughly 10- to 100-fold lower than those of 5hmC [4,5]. In ES cells, TET1 and TET2 are highly expressed and considered to play roles in reprogramming 5mC and control of the differentiation potential [6,7]. 5fC levels dramatically decrease with ongoing differentiation, suggesting its potential involvement during epigenetic reprogramming [5]. Indeed, immunostaining of zygotes that undergo global demethylation has shown that the appearance of 5fC and 5caC in the male pronucleus is associated with Tet3-mediated loss of 5mC [8].

Bisulfite treatment and subsequent high throughput sequencing (BS-Seq) has been the gold standard for the detection of cytosine methylation. This method, however, does not distinguish 5mC from 5hmC or cytosine from 5fC and 5caC. Specific antibodies have been used to enrich and map 5mC (methylated DNA immunoprecipitation (MeDIP)-Seq) and 5hmC (hydroxymethylated DNA immunoprecipitation (hMeDIP)-Seq) [9]. The use of chemical labeling is an alternative method to enrich and sequence 5hmC in the genome [10,11]. The most recent breakthrough in this field came with two new methods allowing the measurement of 5hmC at single base resolution [12,13]. While various techniques for genome-wide analysis of 5mC and 5hmC are available, there is currently no method that allows the positional mapping of 5fC in the genome. We set out to develop and apply an approach to profile the incidence of 5fC in ES cells by deep sequencing in order to study its involvement in the epigenetic control mechanism.

## Results and discussion

### Development of the 5fC pulldown method

As mass spectrometry analysis has shown 5fC to be present at only 0.02 to 0.002% of all cytosine species in ES cells (approximately 0.12 picomol in 20 µg DNA) [4], we aimed to develop a robust and sensitive method capable of detecting a single 5fC modification within a short (approximately 100 bp) DNA fragment. We considered two independent approaches.

**Figure 1:**
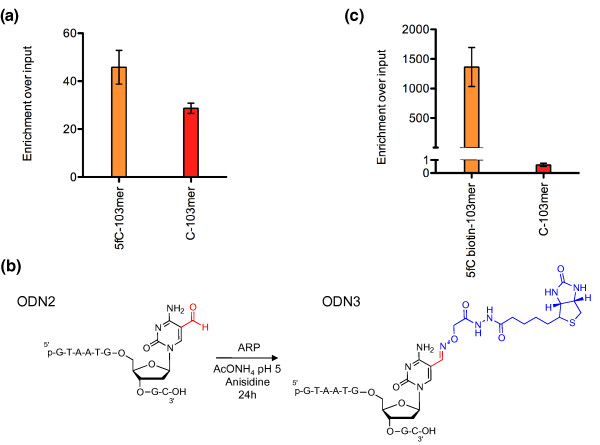
**Comparison between the 5fC-antibody immunoprecipitation and chemical pulldown method**. **(a) **For the 5fC DNA immunoprecipitation we used 1 pg of 5fC-103mer and 10 pg C-103mer in the presence of 5 µg salmon sperm DNA. The immunoprecipitation resulted in an enrichment of 1.6-fold of the 5fC-103mer over the C-103mer. Error bars represent the standard error of the mean. **(b) **Conditions for the biotinylation reaction of a 9-mer containing a single 5fC. The oligonucleotide was incubated at room temperature with an ARP in the presence of anisidine at pH 5 for 24 h and resulted in the formation of a single product. **(c) **Pulldown of 1 pg 5fC-biotin-103mer in the presence of 10 pg C-103mer and 5 µg salmon sperm DNA using streptavidin-coated magnetic beads resulted in an enrichment of the biotinylated DNA of around 1,000-fold. Error bars represent the standard error of the mean.

The first method used a commercially available 5fC antibody that had previously been used to stain 5fC during mouse pre-implantation development [8]. We evaluated the suitability of this antibody for immunoprecipitation using two synthetic 103-mers, each bearing only a single CpG motif, one with and one without a 5fC modification, as a stringent model system (Table s1 in Additional file 1). 5fC-DNA pulldown (5fC-DP) analysis revealed that the antibody showed only 1.6-fold discrimination between the 5fC-positive and 5fC-negative DNA strands (Figure 1a).

The second approach involved the chemical reaction of 5fC with an oxyamine functionality to covalently attach the biotin tag and subsequent pulldown using streptavidin-coated magnetic beads. We optimized the oxyamine reaction by adapting the conditions first reported by Pfaffeneder *et al*. [5]. The oligonucleotide was incubated with a commercially available aldehyde reactive probe (ARP; O-(biotinylcarbazoylmethyl) hydroxylamine) in the presence of anisidine at pH 5 for 24 h at room temperature. We used mass spectrometry to monitor the reaction between a synthetic 9-mer oligonucleotide containing all the four bases (G, A, T and C) plus a single 5fC modification, and the ARP (Figure 1b; Figure s1A and Table s2 in Additional file 1). After 24 h, the formation of a single biotinylated oligonucleotide was observed without side products. We tested the same reaction on a synthetic oligomer where 5fC was replaced by 5hmC. This reaction led exclusively to the recovery of starting material demonstrating very high specificity for the biotin labeling reaction (Figure s1B in Additional file 1). We then optimized pulldown conditions (see Materials and methods) using the biotinylated 103-mer to achieve enrichments of around 1,000-fold for the 5fC-containing DNA (Figure 1c). Thus, in our hands, the chemical pulldown method exhibited considerably higher 5fC-DNA enrichment than the antibody-based immunoprecipitation and was therefore employed for the genome-wide mapping of 5fC. We also tested the selectivity of our method for the pulldown of 5fC versus abasic sites (apurinic/apyrimidinic sites) as ARPs have previously been used to tag abasic sites [14]. We found that our pulldown method was highly specific for the enrichment of 5fC (Figure s2 and Table s3 in Additional file 1).

### Genome-wide mapping of 5fC

We adapted a published method for the pulldown of biotinylated DNA [11], to analyze the distribution of 5fC in ES cells by deep sequencing of enriched DNA fragments.

**Figure 2:**
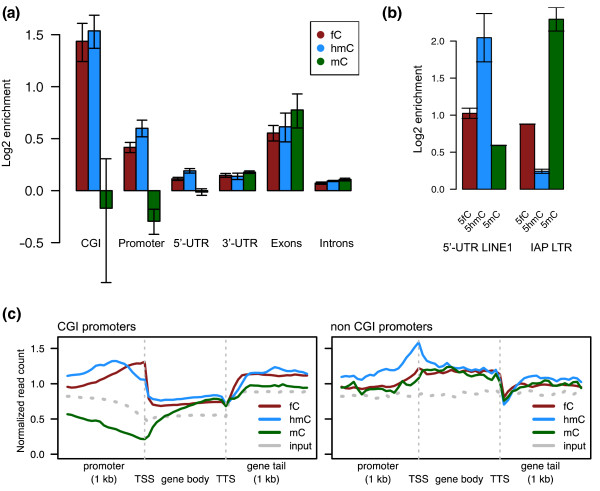
**Genomic distribution of 5fC in comparison to 5hmC and 5mC**. **(a) **Average enrichment (log2 of read count in pull down/read count in input) of 5fC, 5hmC, and 5mC in different genomic regions. 5fC and 5hmC followed similar distributions with high enrichments in CGIs, promoters and exons. Error bars are the mean of each replicate. **(b) **Distribution of 5fC, 5hmC and 5mC in the 5' UTR of retrotransposon elements LINE1 and in IAP LTR. Error bars are the mean of each replicate. **(c) **Genes were classified into CGI- and non-CGI-containing promoters and the normalized read count (reads per kilobase per million mapped reads (RPKM)) averaged across all genes. The profile of genes with CGI promoters showed high levels of 5hmC and 5fC close to the transcription start site (TSS), while an opposite trend was observed for 5mC. In contrast, genes with non-CGI promoters showed low levels of all three cytosine modifications in the promoter. The levels increased slightly near transcription initiation sites and then stayed constant throughout the gene body.

Sequencing libraries were tested for the efficiency of the pulldown step by quantitative PCR of the synthetic oligonucleotides 5fC-103mer (1 pg) and C-103mer (10 pg) that were spiked into the sample prior to pulldown. Samples were sequenced in two biological replicates, which were found to be highly reproducible in terms of correlation between replicates at the significantly enriched sites (Figure s3 in Additional file 1). A genomic input library was also prepared and sequenced and was used as background control for the identification of read-enriched regions in the pulldowns. We compared the 5fC data with published MeDIP-Seq and hMeDIP-Seq data [9] for 5mC and 5hmC, respectively. It is noteworthy that similar to MeDIP-Seq and hMeDIP-Seq, 5fC-DP-Seq shows the relative enrichment to the input library rather than the absolute 5fC levels. TET1 binding sites (data taken from [15]) were enriched in 5hmC and 5fC, but not 5mC, which is in accordance with the fact that TET1 is the catalyst for the generation of 5hmC and 5fC (Figure s4 in Additional file 1). The genome-wide distribution of 5fC followed a similar pattern to 5hmC with enrichments in euchromatic regions, including CpG islands (CGIs), exons and promoters (Figure 2a; Additional file 2). We also looked at the 5' UTR of LINE1 and the intracisternal A particle long terminal repeat (IAP LTR), all of which showed enrichments of 5fC in contrast to the depletion in the gene body of LINE and also other retrotransposon elements (Figure 2b; Additional file 1, Figure s5). The 5' UTR of LINE1 displayed high levels of 5hmC, medium levels of 5fC and low levels of 5mC. In contrast, IAP LTR had low levels of 5hmC, medium levels of 5fC and high levels of 5mC, demonstrating that the kinetics at each oxidation stage depends on the genomic context. It remains to be seen if these patterns are associated with active demethylation.

The profiles shown in Figure 2c represent the enrichment levels of cytosine modifications for all genes separated into CGI- or non CGI-containing genes. In CGI-containing promoters there is a sharp enrichment peak of 5fC at the transcription start site and a slightly less localized enrichment of 5hmC with a depletion of 5mC at the transcription start site. In contrast, the profile of non-CGI promoter regions of the reference genes showed a much less pronounced increase in the levels of both 5mC and 5fC upstream of the transcription start site; these then remain at a constant level throughout the gene bodies. Overall, our analyses show that, depending on genomic regions, we observed different distributions of 5fC, 5hmC and 5mC, which suggests that the kinetics of processing 5mC are distinct between genomic regions. That 5fC is especially enriched in CGIs also supports the role of 5fC in the maintenance of hypomethylation in these regions in ES cells.

### 5fC is associated with active gene expression in ES cells

**Figure 3:**
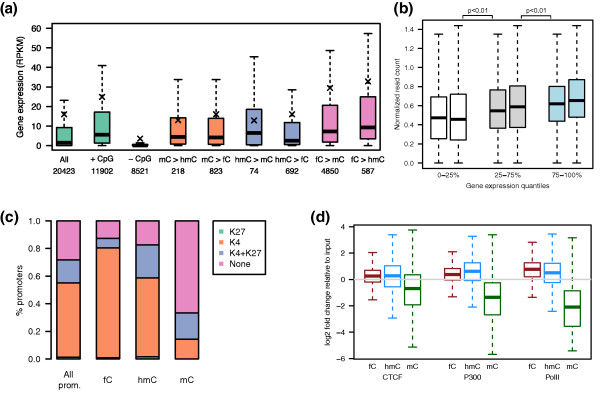
**5fC is associated with active gene transcription**. **(a) **Relationship between gene expression levels and cytosine modifications. The first three green bars show the gene expression levels of all genes (All), genes with CGI promoter (+ CpG) and genes with non-CGI promoters (- CpG). The subsequent bars are labeled with the notation '*x > y*' (for example, *mC > hmC*) to indicate genes whose promoter CGI is relatively more enriched in one modification (for example, *mC*) than in the other (for example, *hmC*). The number below each label is the number of genes belonging to each category. See Table s5 in Additional file 1 for significance of difference between groups. The × symbols show the group means. In all the boxplots in this figure the whiskers extend up to 1.5 times the interquartile range and data points beyond this range have been omitted for clarity of presentation. **(b) **Genes are categorized into low (0 to 25%, white), medium (25 to 75%, grey) and high (75 to 100%, blue) expressed genes and correlated with 5fC levels of two biological replicates (normalized read counts). **(c) **Relationship between 5fC, 5hmC, 5mC and H3K4me3 and H3K27me3 at promoters. **(d) **Enrichments of cytosine modifications in the transcription factor binding sites of CTCF, p300 and Pol II over the input. RPKM, reads per kilobase per million mapped reads.

We identified CGIs that showed a significant difference in 5fC enrichment compared to 5mC and 5hmC, and further characterized them using gene ontology categories. Therefore, we associated each island with the nearest gene within 5 kb and searched for overrepresented categories in this set. Gene ontology analysis of the 5fC-enriched genes identified pathways that were associated with transcription regulation (Table s4 in Additional file 1). We also examined the correlation between 5fC at CGI gene promoters and their transcription levels using published gene expression data [9]. Specifically, we compared gene expression levels for cases where one cytosine modification in the CGI promoter region was relatively more enriched than other cytosine derivatives. We found that genes whose CGI promoters were 5fC-rich (relative to 5mC or 5hmC) showed higher expression than the overall group of CGI assigned genes (Figure 3a; Table s5 in Additional file 1). This suggests that the shift in equilibrium between the different cytosine modifications at promoter sites may be linked to mechanisms that control gene activity. Consistent with this observation, when genes were categorized as low, medium or highly expressed genes, expression levels positively correlated with 5fC levels (Figure 3b). Promoters enriched in 5fC were also associated with correspondingly high levels of the activating histone mark H3K4me3 (histone H3 tri-methylated at lysine 4; data taken from [16]), whereas those enriched in 5mC were depleted of H3K4me3 (Figure 3c). When we measured the enrichment of cytosine modifications in the binding sites of three key transcription regulatory elements, CTCF, p300 and RNA polymerase II (Pol II), we found that 5fC was significantly enriched in Pol II binding sites, supporting a strong link between this modification and gene regulation (Figure 3d; Figure s6 in Additional file 1). In contrast, we observed low levels of 5fC in binding sites of CTCF, a transcriptional repressor. Collectively, these data associate the presence of 5fC with transcriptional activity of genes in ES cells. It remains to be seen whether 5fC represents a mark that is poised for rapid demethylation of incoming aberrant methylation in CGIs and is thus linked to the onset of gene activation or indeed whether 5fC on its own is involved in epigenetic control mechanisms.

### TDG knockdown results in genomic redistribution of 5fC

TDG was initially shown to mediate the base excision repair of deaminated 5mC. Recent studies have shown that TDG can also excise 5fC and 5caC, providing a new dimension to our understanding of the active demethylation pathway [3,17]. To further understand the role of TDG, we analyzed the changes in the genomic distribution of 5fC in ES cells depleted for TDG, compared to a control in which cells were treated with a non-targeting small interfering RNA (siRNA). The knockdown (KD) resulted in the downregulation of TDG expression by 97% (Figure s7 in Additional file 1). Mass spectrometry analysis showed that TDG KD increased overall 5fC levels by six-fold, consistent with its role in excising 5fC, whereas methylation levels stayed constant (Figure s8 in Additional file 1). In general, we found that more than 98% of 5fC-enriched regions from TDG KD overlapped with those found in the siRNA control. Genome-wide 5fC mapping of the TDG KD showed 5fC-enriched sites were distributed with a reduced overall coverage of the genome (5fC sites distributed over 138 Mb in contrast to 415 Mb in the control). Thus, 5fC must be present at higher levels and/or higher density in the enriched sites for the TDG KD. This also indicates that the formation of 5fC marks at those remaining 277 Mb must be via a distinct pathway that is TDG-dependent, perhaps involving TET recruitment by TDG. It can also mean that the loss of 5fC in these particular regions is TDG-independent via an alternative pathway.

**Figure 4:**
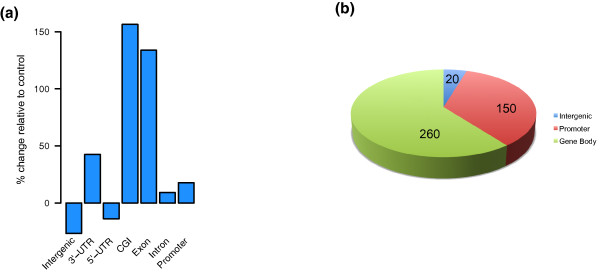
**Effect of TDG knockdown on 5fC distribution**. **(a) **Percentage change of annotated regions between TDG and siRNA control. Regions affected in the TDG KD (enriched in 5fC in TDG KD relative to the siRNA control) were mostly assigned to CGIs and exons. **(b) **CGI found in regions enriched in TDG knockout relative to siRNA control classified by genomic annotation.

We then compared the enriched regions between TDG KD and siRNA control and found that 5% (out of 138 Mb) were significantly more highly enriched than in the control. We annotated these regions to genomic functions and found that the change predominantly affected CGIs and exons (Figure 4a). The CGIs were mostly found in gene bodies and, to a certain extent, in promoters (Figure 4b). We identified the top 43 CGIs that showed the biggest increase in 5fC in the TDG-control and assigned it to the nearest gene. Gene ontology analysis of the genes acquiring 5fC revealed a significant link with pathways associated with cell morphogenesis and neural development/differentiation (Table s6 in Additional file 1).

### Lack of 5fC excision in CGIs is associated with aberrant DNA methylation patterns in differentiated cells

**Figure 5:**
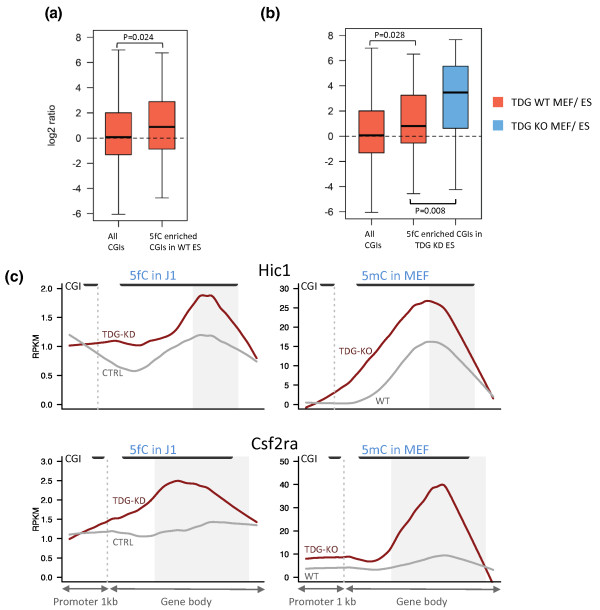
**Effect of TDG knockdown on 5fC distribution**. **(a) **The ratio of methylation was plotted for TDG wild-type (WT) MEFs versus TDG± ES cells for the group of CGIs that show the highest 5fC enrichment in ES cells and a control group of all CGIs. The 5fC-enriched CGIs have higher methylation in the MEFs than the ES cells. **(b) **The ratio of methylation for WT MEFs (in red), or TDG KO MEFs (in blue), versus the ES cells. The 43 CGIs with the biggest increase in 5fC upon TDG KD have higher methylation in MEFs than ES cells and this increase is bigger in the absence of TDG. **(c) **Two example profiles (Hic1 and Csf2ra) show the overlap between 5fC CGI peaks in TDG KD ES cells and 5mC peaks at CGIs in TDG KO MEFs. The dotted grey line marks the transcription start site whereas the solid black lines represent CGIs. The shaded grey areas are the regions that are enriched for 5fC over control. RPKM, reads per kilobase per million mapped reads.

Next, we considered the role of 5fC in cells that were derived from developing mice that lacked TDG (TDG knockout (KO)). We hypothesized that lethality of TGD KO mice during embryogenesis (embryonic day 11.5) could be attributed in part to the inability to excise 5fC in the genome, which might interfere with lineage commitment of ES cells during differentiation. Differentiation of TDG KO ES cells to neuronal progenitor cells was shown previously to lead to hypermethylation of promoter CGIs in genes that include homeobox transcription factors and accumulation of DNA methylation at these sites was even more pronounced in mouse embryonic fibroblasts (MEFs) derived from TDG KO mice [18]. We therefore identified the top 5fC-enriched peaks in ES cells and compared them to MeDIP-Seq datasets for wild-type and TDG KO embryonic fibroblasts (MEFs) and heterozygous ES cells (TDG± ES cells) from the HEROIC consortium [19]. We found that CGIs overlapping these regions showed a tendency to gain methylation during development (Figure 5a). Our data indicate that the presence of 5fC at a CGI may promote its methylation during differentiation. The increase in methylation from ES cells to MEFs was most pronounced for the 43 CGIs that showed the largest increase in 5fC upon TDG KD (Figure 5b). Moreover, the gain in methylation at these TDG-target CGIs is even larger in the absence of TDG, in MEFs derived from TDG KO embryos (Figure 5b and examples in Figure 5c). This suggests that the increased 5fC at these CGIs resulting from the absence of TDG in the pluripotent stage of the early embryo may promote an even higher gain of methylation during development. In addition, TDG may also be acting in complementary pathways at these target CGIs to remove excess DNA methylation - for example, repairing mismatches resulting from 5mC deamination.

We also analyzed the 5fC distribution following siRNA-mediated down-regulation of TET1, which led to a 50% decrease in genomic 5fC as measured by mass spectrometry (data not shown). Due to the presence of TET2 in ES cells, which presumably overlaps with TET1 in binding to chromatin in many genomic regions, we concluded that additional work is needed to interpret the potential interaction between TET1, TET2 and TDG in ES cells, which is beyond the scope of this study.

## Conclusions

We have developed a robust and sensitive method to chemically label 5fC in genomic DNA. We have used this technique to enrich 5fC-containing DNA and have studied the genome-wide distribution of 5fC in mouse ES cells. Our study showed that 5fC was enriched in CGIs, which supports the role of 5fC in the maintenance of hypomethylation at CGIs in ES cells. CGI promoters that were more enriched in 5fC levels than 5hmC or 5mC correlated with active gene expression. Furthermore, 5fC-enriched promoter regions overlapped with H3K4me3 marks and 5fC was found enriched in the binding sites of Pol II. Overall, these observations suggest that the presence of 5fC marks is correlated with active gene expression in ES cells. TDG KD data demonstrated that TDG is actively involved in the removal of 5fC marks in CGIs, exons and promoter regions. This is in agreement with previous observations that TDG is involved in the maintenance of epigenetic stability by protecting CGIs from hypermethylation [18,20]. We also found that CGIs that showed the largest increase in 5fC upon TDG KD became methylated during normal differentiation, suggesting that 5fC excision may be necessary for the establishment of correct methylation pattern during differentiation.

## Materials and methods

### General methods

Commercial reagents were used as received unless otherwise noted. ARP (O-(biotinylcarbazoylmethyl) hydroxylamine))was purchased from Cayman Chemical(Ann Arbor, Michigan, USA), Alexa Fluor® 488 hydroxylamine from Invitrogen (Carlsbad, California, USA), ammonium acetate from Sigma (Dorset, UK), anisidine from Aldrich (Dorset, UK)and potassium perruthenate from Alfa Aeasar (Ward Hill, Massachusetts, USA). Acetonitrile for high performance liquid chromatography (HPLC)-electrospray ionization (ESI)-mass spectrometry (MS) analysis was purchased from VWR (Radnor, Pennsylvania, USA), HPLC gradient grade. NHEt_3_OAc for HPLC buffers was purchased from Glenn Research (Sterling, Virginia, USA).

### HPLC-ESI-MS analysis of oligonucleotides

The samples were analyzed by HPLC-ESI-MS on a Bruker (Fremont, USA) amaZon × Ion Trap MS and chromatographed by a Dionex (Sunnyvale, CA, USA ) UltiMate 3000 UHPLC system equipped with a diode array detector and a column oven. HPLC analysis of oligonucleotides was performed on a Nucleosil C18 column (250 × 4.6 mm, 5 μm; Macherey Nagel (Dueren, Germany) using the following solvent system: solvent A, 50 mM NHEt_3_OAc pH 7.4; solvent B, CH_3_CN; flow rate of 1 ml.min^-1^; a linear gradient of 0 to 30% was applied over 20 min. The column temperature was maintained at 30°C. The elution was monitored at 260 and 280 nm (Dionex UltiMate 3000 Diode Array Detector). Ions were scanned by use of a negative polarity mode for oligonucleotides.

### Mass spectrometry of nucleosides

Genomic DNA was digested using DNA Degradase Plus (Zymo Research, Irvine, CA, USA) according to the manufacturer's instructions and analyzed by liquid chromatography-tandem mass spectrometry on a LTQ Orbitrap Velos mass spectrometer (Thermo Scientific, Waltham, Massachusetts, USA) fitted with a nanoelectrospray ion-source (Proxeon, Odense, Denmark). Mass spectral data for 5fC and 5mC were acquired in high resolution full scan mode (R >40,000 for the protonated pseudomolecular ions and >50,000 for the accompanying protonated base fragment ions). Data for 5mC were also acquired in selected reaction monitoring (SRM) mode monitoring the transition 242 → 126.0662, with HCD fragmentation using a 4 mass unit parent ion isolation window, a relative collision energy of 20% and R >14,000 for the fragment ions. Peak areas for the 5fC and 5mC fragment ions were obtained from extracted ion chromatograms of the relevant scans.

### Synthesis of ODN2 (oxidation of 5hmC containing oligonucleotide ODN1)

Oxidation of 5hmC containing oligonucleotide ODN1 was performed by adapting a procedure reported by Booth *et al*. [12]. The oxidation was performed on a 5'-phosphate protected oligonucleotide to avoid any side reactions due to the oxidation of the 5' primary hydroxyl group. 5hmC containing oligonucleotide ODN1 (1.2 nmol, 8 μM, 1 eq) and KRuO_4 _(90 nmol, 600 μM, 75 eq) were mixed in a 50 mM NaOH aqueous solution and placed on ice for 15 min. The reaction was stopped by standard ethanol precipitation. The calculated and found masses of ODN2 are reported in Table s2 and Figure s1A in Additional 1.

### Synthesis of ODN3 (biotin-labeling of fC containing oligonucleotide ODN2)

The biotin conjugate was obtained by adapting a procedure reported by Pfaffeneder *et al*. [5]. 5fC containing oligonucleotide ODN2 (0.27 nmol, 8 μM, 1 eq) in a 40 mM aqueous NH_4_OAc buffer pH 5.0 supplemented with 100 mM anisidine was incubated with N-(aminooxyacetyl)-N'-(D-biotinoyl) hydrazide) (13.6 nmol, 400 μM, 50 eq) for 24 h at 25°C. The reaction was stopped by standard ethanol precipitation. Figure s1A in Additional file 1 shows the LC-MS analysis of the product ODN3 and the calculated and found masses are reported in Table s2 in Additional file 1.

In order to control the specificity of the reaction, the same reaction was carried out on ODN1; in the absence of the oxidation step, only the starting material was recovered (Figure s1B in Additional file 1).

### Biotin-labelling of fC in genomic DNA samples

Genomic DNA was prepared by sonicating genomic DNA extracted from mouse embryonic stem cells J1. Genomic DNA was sonicated for 4 × 15 cycles (30 s on, 30 s off pulse) using a Diagenode Bioruptor sonicator in order to obtain 200 to 500 bp fragments. The incubation of genomic DNA samples with ARP was carried out in analogy with the procedure for synthetic oligonucleotides. The final DNA concentration was adjusted to 5 ng/μl in a 40 mM aqueous NH_4_OAc buffer pH 5.0 supplemented with 100 mM anisidine and 2 mM ARP. After the reaction, the DNA was purified using the GeneJet PCR purification kit (Fermentas, Waltham, Massachusetts, USA) and eluted in 50 µl elution buffer (10 mM Tris-HCl, pH 8.5).

### Labeling of deoxyribose and oligonucleotide

The biotin conjugates were obtained by adapting a procedure reported by Ide *et al*. [14]. Therefore, 2-deoxyribose 5-phosphate or abasic sites containing 103-mer (100 μM) was incubated with ARP (2 mM) in phosphate-buffered saline (pH 7) for 1 h at 37°C.

### Synthesis of abasic sites containing 103-mer

The abasic sites containing 103-mer were obtained in 2 steps starting from the incorporation of uracil into 103-2 DNA using the Dreamtaq polymerase (Fermentas) (Table s1 in Additional file 1). The DNA was then purified using the GeneJet PCR purification kit. Subsequent treatment of the uracil-containing oligonucleotide with Uracil-DNA-Glycosylase (NEB, Ipswich, Massachusetts, USA) afforded the abasic sites containing 103-mer.

### Pulldown experiment and Illumina library preparation

The antibody pulldown experiment was done following a procedure reported by Ficz *et al*. [9]. For the chemical pulldown, the ends of the DNA fragments were repaired and paired-end sequencing specific adaptors (Illumina, San Diego, California, U.SA) were ligated using the NEBNext DNA Sample Prep Reagent Set 1 (NEB). Following adaptor ligation, DNA and 2 μg poly-dI:dC were incubated with 50 μg streptavidin coated magnetic beads (MagneSphere Promega, Fitchburg, Wisconsin, USA) in 50 μl 2× binding buffer (10 mM Tris pH 7.5, 1 mM EDTA, 2 M NaCl, 0.1% TWEEN) for 15 minutes at room temperature. Beads were washed with 5× 500 μl binding buffer and transferred into a new eppendorf. For elution, beads were incubated with 100 μl elution solution (95% formamide, 10 mM EDTA and 400 nM biotin) at 90°C for 5 minutes and the eluant was collected and immediately placed on ice. This step was repeated to elute any residual DNA. Eluted DNA was then precipitated in ethanol and the DNA pellet was resuspended in 15 μl *dd*H_2_O. Fragments were amplified with 16 cycles using adaptor specific primers (Illumina); fragments ranging between 200 and 500 bp in size were gel purified before cluster generation and sequencing. Sequencing was done on an Illumina Genome Analyzer GAIIX using Cluster Generation v4 and 5 chemistries as well as Sequencing by Synthesis Kit v5. Data collection was performed using Sequencing Control Software v2.6 and 2.9. Real-time Analysis (RTA) 1.6 and 1.9 were used for base calling.

### Enrichment test by quantitative PCR prior sequencing

Before each pulldown, genomic DNA was spiked with two synthetic oligomers: 1 pg of 103 bp DNA containing one single biotin-fCpG and 10 pg of 103 bp DNA with one single CpG. C-103mer was added 10-fold more than fC-103mer in order to get similar Ct values for both strand amplification. Details on the sequence and primers used are given in Table s1 in Additional file 1. After pulldown, enrichment was validated by quantitative RT-PCR.

### Culturing of ES cells and RNA interference knockdown of Tet1 and Tdg

Cell culturing was done on J1 ES cells line (129S4/SvJae), purchased from ATCC (catalogue number SCRC-1010) and grown on a γ-irradiated pMEF feeder layer at 37°C and 5% CO_2 _in complete ES medium (DMEM 4,500 mgl^-1 ^glucose, 4 mM L-glutamine and 110 mg l^-1 ^sodium pyruvate, 15% fetal bovine serum, 100 U of penicillin/100 mg of streptomycin in 100 ml medium, 0.1 mM non-essential amino acids, 50 mM β-mercaptoethanol, 10^3^U LIF ESGRO). RNA interference experiments were performed in J1 ES cells without feeders with three rounds of transfections with siRNA every second day. In the first day 1 × 10^5 ^cells were seeded per well (3.8 cm^2^) of a 12-well plate and adherent cells were transfected the next day with 50 pmol siRNA: 3 μl Lipofectamine™2000 complexes in media without antibiotics according to the manufacturer's instructions. After 8 h fresh media with antibiotics was added to the cells. This procedure was repeated 48 and 96 h after the first transfection, both times on cells in suspension. Cells were passaged and transfections were scaled up when necessary. Transfections were done with Dharmacon (Lafayette Colorado, USA) siGENOME siRNA duplexes (Thermo Fisher Scientific, Waltham, Massachusetts, USA) against mouse Tet1 (catalogue number D-062861-01; caacuugcauccacgauua), siGENOME SMARTpool against mouse Tdg (catalogue number M-040666-01; gaagugcaguauacauuug, gaguaaagguuaagaacuu, caaagaagauggcuguuaa, gcaaggaucugucuaguaa) and siGENOME non-targeting siRNA#2 (catalogue number D-001210-02; sequence not available). Cells were harvested after three rounds of transfection for DNA/RNA isolation.

### Bioinformatics and data analysis

Reads in fastq format obtained from the Illumina sequencing pipeline have been aligned against the mouse genome (NCBI version mm9) using bwa [21] with default settings. Before further analyses, only reads unequivocally assigned to a single genomic position (that is, reads mapped with mapq quality of 15 or greater) were retained (see Table s7 in Additional file 1 for details of total number of reads and mapped reads). The libraries enriched for 5hmC and 5mC were downloaded from the Short Read Archive (accession ID ERP000570; run IDs ERR031631 and ERR031628 for 5hmC; run IDs ERR031630 and ERR031627 for 5mC). These libraries were processed as above, although only the first mate of each pair and only the first 40 bases of each read were analyzed in order to conform them with the 5fC sequencing protocol (single-end, 40 cycles).

Genomic regions enriched in 5fC, 5hmC or 5mC were identified with the program rseg [22] in mode 2 using the input library as control and setting the bin size to 100 bp. Each replicate was analyzed separately and a consensus enrichment was compiled by intersecting the enriched regions from the two replicates of each treatment. The consensus regions are provided as supplementary files (Additional file 2). The regions affected by the Tet1 or Tdg KD - that is, the regions enriched in 5fC in the KD relative to the control KD - were identified by running rseg in mode 3 with bin size of 100 bp (Additional file 2).

The position of the functional regions (CGI, intron, exons, and so on) as well as the position of CTCF, p300 and Pol II were extracted from the UCSC genome browser [23].

The identification of CGI relatively more enriched in one modification over another was performed by assuming that the number of reads overlapping each CGI follow a negative binomial distribution. The difference between conditions was tested by means of the exact test described in Robinson *et al*. [24]. These analyses were performed using the R/Bioconductor package edger [25]. All the analyses not mentioned above were performed by means of samtools [26], bedtools [27] and custom python and R scripts. MeDIP-seq data for the TDG KO analysis from the HEROIC Consortium (accession ID GSE27468) were aligned with Bowtie in paired-end mode using options -m 1 --best --strata --maxins 700 --chunkmbs 512. Gene ontology annotation was executed via the web service DAVID v6.7 [28] by means of the Functional Annotation tool [29].

### Data access

The raw sequence data used to map 5fC and the position of the 5fC enriched regions for each library have been deposited at NCBI Gene Expression Omnibus under accession number GSE40148.

## Abbreviations

5caC: 5-carboxylcytosine; 5fC: 5-formylcytosine; 5hmC: 5-hydroxymethylcytosine; 5mC: 5-methylcytosine; ARP: aldehyde reactive probe; bp: base pair; CGI: CpG island; DP: DNA pulldown; ES: embryonic stem; ESI: electrospray ionization; H3K4me3: histone H3 tri-methylated at lysine 4; hMeDIP: 5-hydroxymethylcytosine DNA immunoprecipitation; HPLC: high performance liquid chromatography; IAP LTR: intracisternal A particle long terminal repeat; KD: knockdown; KO: knockout; LINE: long interspersed element; MeDIP: 5-methylcytosine DNA immunoprecipitation; MEF: mouse embryonic fibroblast; MS: mass spectrometry; Pol II: RNA polymerase II; siRNA: small interfering RNA; TDG: thymine DNA glycosylase; TET: ten-eleven translocation; UTR: untranslated region.

## Competing interests

The authors declare that they have no competing interests.

## Authors' contributions

EAR designed and performed the study and wrote the manuscript; DB performed data analysis and provided feedback on the manuscript; GF assisted with study design, carried out sample preparation and provided feedback on the manuscript; HB carried out sample preparation, assisted with data analysis and provided feedback on the manuscript; MB assisted with data analysis and provided feedback on the manuscript; PM assisted with sample analysis and provided feedback on the manuscript; DO assisted with sample analysis; MJB assisted with sample preparation; WR and SB conceived of the study and edited the manuscript. All authors have interpreted the data, read and approved the manuscript.

## Supplementary Material

Additional file 1 **Supplementary figures and tables**. Click here for file

Additional file 2 **Genomic coordinates of 5fC-, 5hmC- and 5mC-enriched regions in wild type and knockdown**. These bed files report the genomic positions of the regions enriched in 5fC, 5hmC and 5mC. The reported regions are the intersection of the enrichment between two replicates. For each region, the position of the nearest CGI is also shown (where multiple CGIs have the same distance, only the first one is reported). The columns in these files are: 1) chrom (chromosome); 2) start (enriched region start); 3) end (enriched region end); 4) name (ID for enriched region); 5, value (unused column); 6) strand (strand (always +)); 7) cgi_start (nearest CGI start); 8, cgi_end (nearest CGI end); 9) cgi_id (CGI ID (from UCSC genome browser table cpgIslandExt.bed)); 10) cgi_dist (distance of CGI from enriched region (0 if overlapping)). The files in the zipped directory are: fc.enriched.cgi.bed (regions enriched in 5fC in wild-type); fc_sirna_ctrl.enriched.cgi.bed (regions enriched in 5fC in siRNA control); fc_tdg_vs_ctrl.enriched.cgi.bed (regions enriched in 5fC in TDG KD relative to siRNA control); hmc.enriched.cgi.bed (regions enriched in 5hmC wild-type); mc.enriched.cgi.bed (regions enriched in mC wild type). Click here for file
