# Supplementary material for: Genome-wide distribution of 5-formylcytosine in embryonic stem cells is associated with transcription and depends on thymine DNA glycosylase
Source: Genome Biol. 2012 Aug 17;13(8):R69. doi: 10.1186/gb-2012-13-8-r69 (PMC3491369; doi:10.1186/gb-2012-13-8-r69)
Supplement: Additional file 1 — Supplementary figures and tables. [file gb-2012-13-8-r69-S1.DOCX]

# Additional file 1

**Figure s1. HPLC and EI-MS analysis of ODN1 (B), ODN2 (A) and ODN3 (A).**


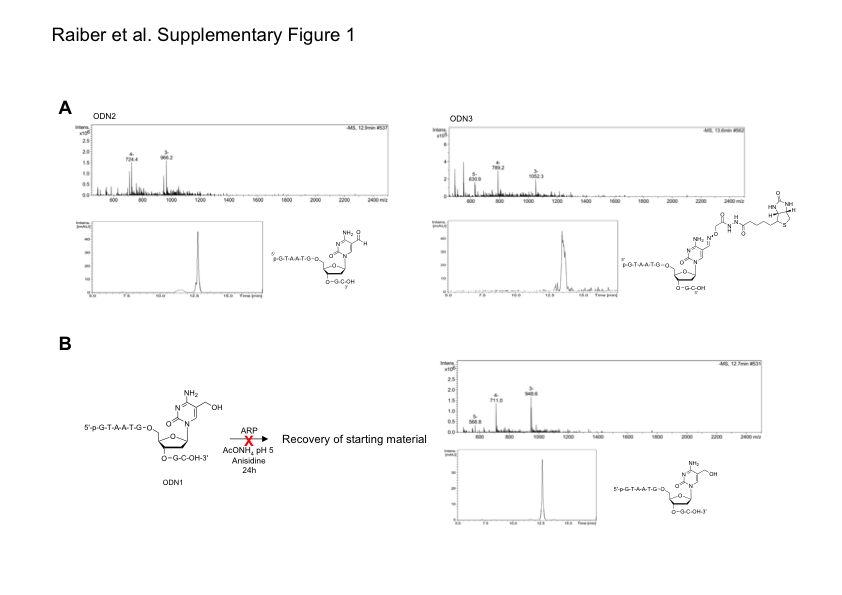


**Figure s2. 5fC versus abasic site enrichments.** A) The mass spectral analysis shows that ARP in PBS (pH7, 37°C, 1h) selectively reacts with deoxyribose, the product of apurinic/ apyrimidinic sites. B) Fluorescent ARP was used to show that the reaction was also selective in double stranded 103-mer containing abasic sites **2** but not 103-mer containing 5fC **1**. C) J1 DNA was prepared for library preparation/ pulldown using both biotinylation conditions (pH5, anisidine, 25°C, 24h and pH7, 37°C, 1h). Subsequent qPCR of seven CGI regions showed that the enrichment observed by sequencing was due to 5fC and not abasic site pulldown. Abasic site pulldown, however, might account for the general background noise.

**
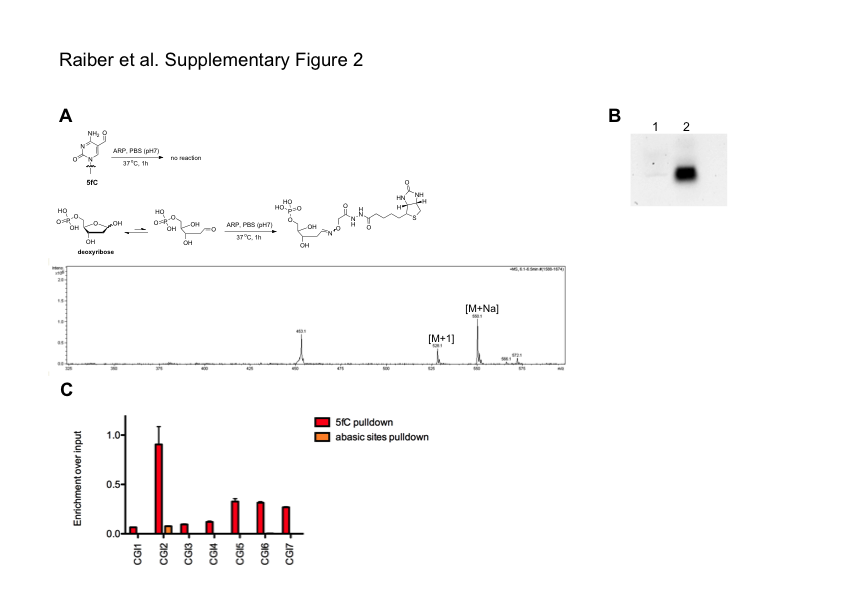
**

**Figure s3. Scatter plot of the two biological replicates of J1 and TDG knockdown.** The plot shows the number of reads (log2) of all enriched regions in replicates bham470 and bham359_362_j1_fc and ear003 and bham480.


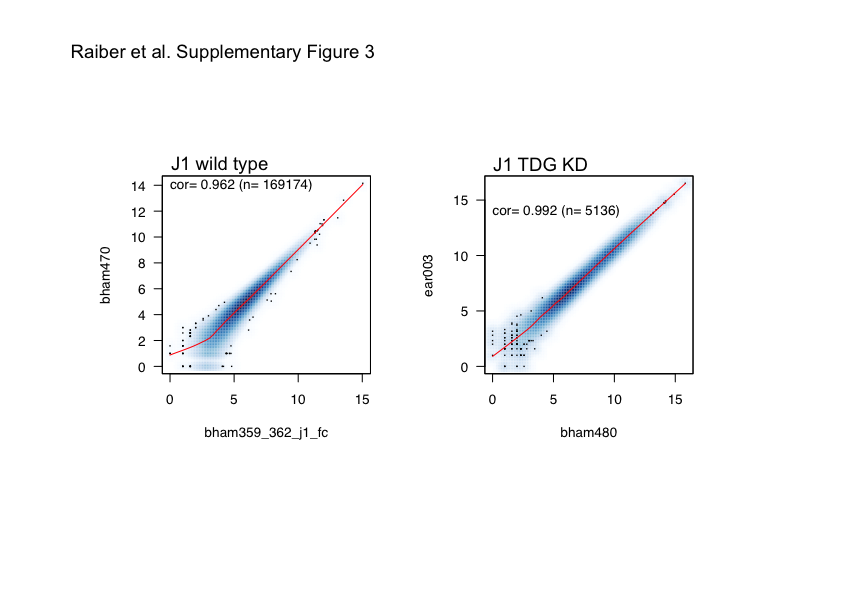


**Figure s4. Link between TET1 binding sites and the cytosine modifications.** The light blue bar shows the log2-fold enrichment of 5fC in TET1 bindings sites whereas the dark blue bar represents the enrichment of 5hmC and the green bar of 5mC.

**
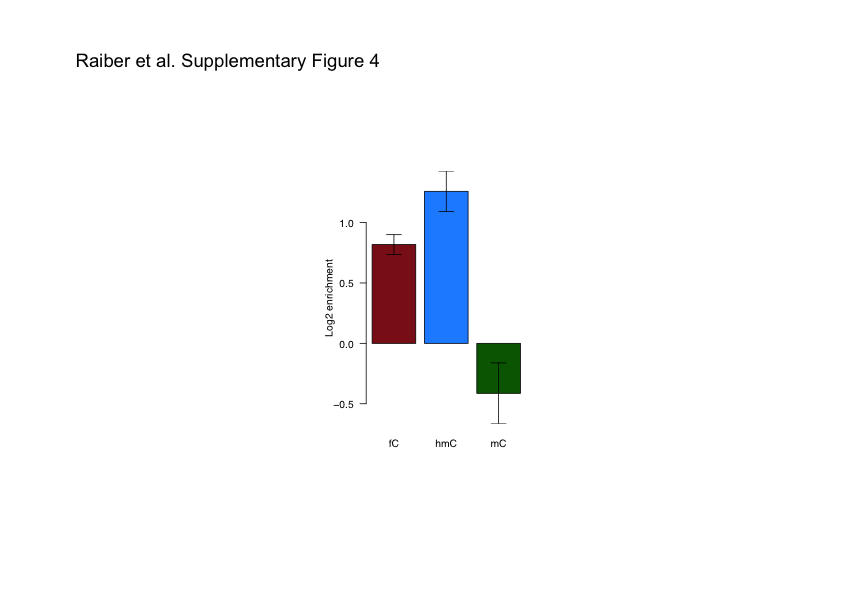
**

**Figure s5. 5fC enrichments in retrotransposon elements.** All three cytosine modifications are low or completely depleted in the retrotransposon elements LINE, SINE and LTR.

**
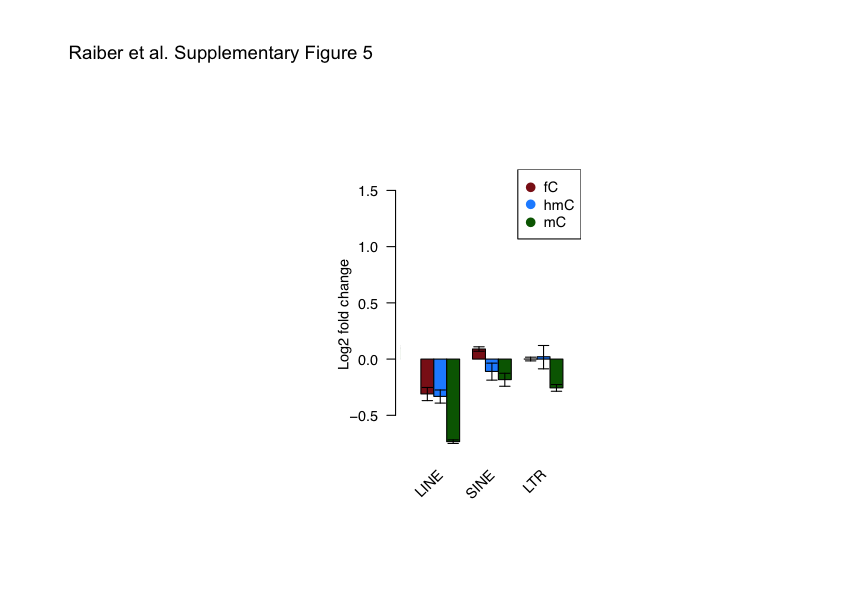
**

**Figure s6. 5fC enrichments in Pol II binding sites.** Enrichment levels of the three cytosine modifications in Pol II binding sites overlapping CGIs and non overalapping CGIs.


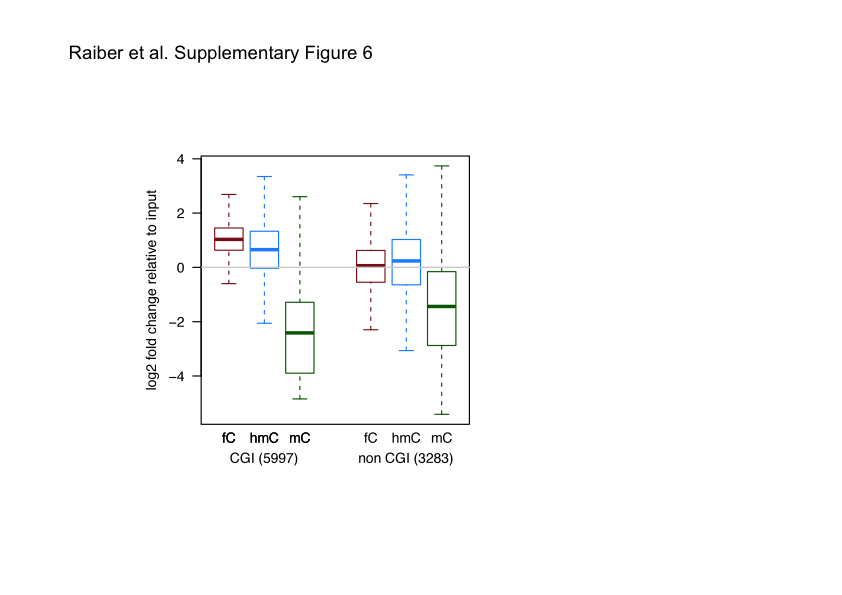


**Figure s7. TDG knockdown efficiency.** Relative TDG expression after treatment of J1 cells with non-targeting siRNA and TDG siRNA (two biological replicates of each).


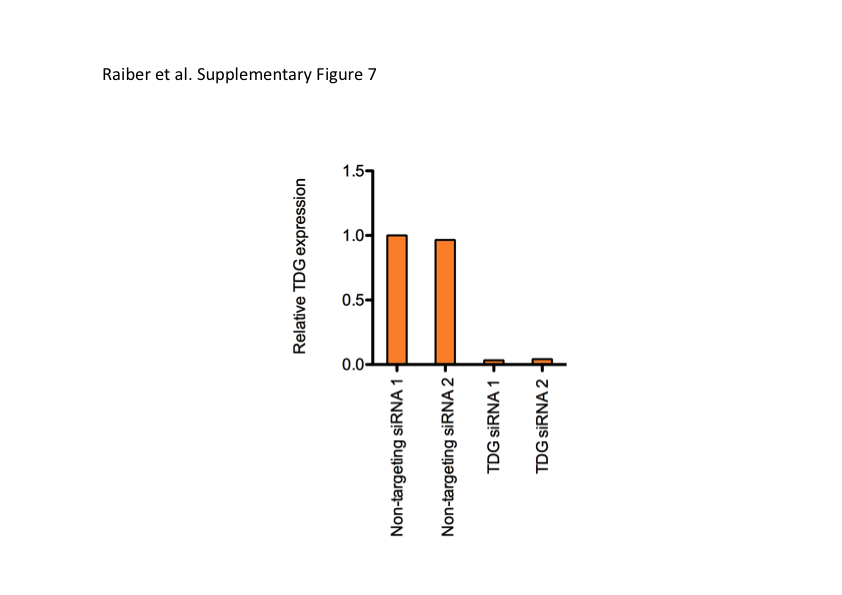


**Figure s8. Mass spectral analysis of knockdown samples.** The graph represents mass spectral data of two biological replicates of siRNA control and TDG knockdown. Three measurements were made for each of the replicates. Levels of 5fC were normalized to 5mC levels.


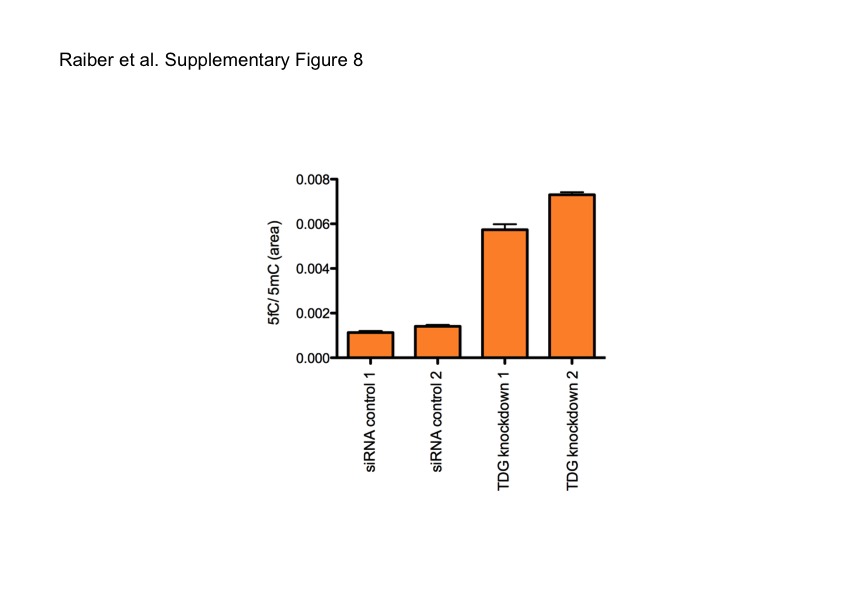


**Table s1. Sequence of 103-mer and primers used for enrichment tests**

| **5fC-103mer** | **C-103mer** | **103-2mer** | **Primer fwd**  **5fC-103mer and 103-2mer** | **Primer rev**  **5fC-103mer and 103-2mer** | **Primer fwd**  **C-103mer** | **Primer rev**  **C-103mer** |
| --- | --- | --- | --- | --- | --- | --- |
| GGAGACTCAGACAGCGAGCGTTTAAATAAATTAAATAATATTAATATATCGATTAATAATAAATAATAATTAATTAATATTCCGTTGACCTTACGATGTCAGG | CAATTCGGAGGAGGCTCGGCTTTAAATAAATTAAATAATATTAATATATCGATTAATAATAAATAATAATTAATTAATATTTAGATCCGCCTTGGAACAGTCG | GGAGACTCAGACAGCGAGCGTCGAATTTCGAACGACGATTAATCGATCGATTAATCGTAACGATTACGTAACGTTCCGTTGACCTTACGATGTCAGG | GGAGACTCAGACAGCGAGCG | CCTGACATCGTAAGGTCAACGG | CAATTCGGAGGAGGCTCGGC | CGACTGTTCCAAGGCGGATCTA |

**Table s2. Sequence and LC-MS characterisation of synthetic oligonucleotides used in this work**

| **Strand N^o^.** | **Sequence** | **Rt (mn)** | **Mass (calc.)** | **Mass (found)** |
| --- | --- | --- | --- | --- |
| **ODN1** | 5’-p-GTA ATG XGC-3’  (X = 5hmC) | 12.7 | 2847.8 | 2847.8 |
| **ODN2** | 5’-p-GTA ATG XGC-3’  (X = 5fC) | 12.9 | 2845.8 | 2845.6 |
| **ODN3** | 5’-p-GTA ATG XGC-3’  (X = 5fC-biotin) | 13.6 | 3159.8 | 3158.9 |

**Table s3. Primers used for qPCR of genomic regions**

|  | **Primer fwd** | **Primer rev** |
| --- | --- | --- |
| **CGI 1** | ACAAAACTCGCAGGCAATC | TAGAACAGCCTTTCCCGTCT |
| **CGI 2** | CCTGCACGTAGGGTTTCC | CTTTCTGCTGGTCACTGAGG |
| **CGI 3** | TCGGGATCCAGAAGTGCATGGGA | TGCCGGGGACTAATGGACTGAG |
| **CGI 4** | GAGCCAGAGCCCGTGGAAGGA | TCTACCAGGGCTGGATGGTGCT |
| **CGI 5** | CGCTCTCTGGCTTCCGCCAC | GGAGCCTAGGGCTCACCACCG |
| **CGI 6** | CGCAGCCAGCACCCACA | TCTGTAGGAAGTGATGGAGGAGC |
| **CGI 7** | GCAACTGTAGGGAGCTAGGG | ACCGTCCTCACCTCCAATC |

**Table s4. GO analysis of 5fC enriched CGIs in J1.** The analysis identified significant enrichment of pathways associated with transcription regulation.

|  | **P value** | **Benjamini** |
| --- | --- | --- |
| **Nucleus** | 2.7E-27 | 6.5E-25 |
| **Transcription regulation** | 5.0E-19 | 6.0E-17 |
| **DNA binding** | 6.8E-19 | 7.0E-16 |
| **Transcription** | 4.2E014 | 4.1E-12 |

**Table s5.** **Significance of difference in expression for the genes presented in Figure 3A.** Each value is the p-value for difference in mean gene expression, corrected for multiple testing (Holm-Bonferroni correction) and with standard deviation pooled across all groups. In boldface the p-values < 0.01.

|  | All genes | CGI genes | non CGI genes | mC > hmC | mC > fC | hmC > mC | hmC > fC | fC > mC |
| --- | --- | --- | --- | --- | --- | --- | --- | --- |
| CGI genes | **1.51E-16** | - | - | - | - | - | - | - |
| non CGI genes | **1.01E-25** | **3.85E-62** | - | - | - | - | - | - |
| mC > hmC | 1 | 1 | 1 | - | - | - | - | - |
| mC > fC | 1 | 1.84E-01 | **6.95E-03** | 1 | - | - | - | - |
| hmC > mC | 1 | 1 | 1 | 1 | 1 | - | - | - |
| hmC > fC | 1 | 2.70E-01 | 1.64E-02 | 1 | 1 | 1 | - | - |
| fC > mC | **2.88E-19** | 6.31E-02 | **6.01E-56** | 2.28E-01 | **3.90E-03** | 1 | **9.01E-03** | - |
| fC > hmC | **2.27E-04** | 6.55E-01 | **7.08E-13** | 1.59E-01 | 1.72E-02 | 1 | 2.43E-02 | 1 |

**Table s6. GO analysis of 5fC enriched CGIs in TDG knockdown.** The top 43 identified 5fC-enriched CGIs in the TDG knockdown were associated pathways regulating cell morphogenesis and neuron development/differentiation.

|  | **P value** | **Benjamini** |
| --- | --- | --- |
| **Cell projection morphogenesis** | 6.5E-3 | 9.0E-1 |
| **Cell part morphogenesis** | 7.5E-3 | 7.3E-1 |
| **Axonogenesis** | 3.9E-2 | 8.6E-1 |
| **Neuron projection morphogenesis** | 4.4E-2 | 8.6E-1 |

**Table s7. Illumina sequencing runs**

| **library_id** | **N. reads** | **N aligned (%)** | **N. mapq** ≥ **15 (%)** | **Enrichment** | **siRNA** |
| --- | --- | --- | --- | --- | --- |
| bham359_362_j1_fc | 62234480 | 59161550 (95.06) | 45852974 (73.68) | 5fC | wild-type |
| bham470 | 35565749 | 34611689 (97.32) | 27426719 (77.12) | 5fC | wild-type |
| bham479 | 37486491 | 36589182 (97.61) | 29208916 (77.92) | 5fC | control |
| ear001_J1_sirna_ctrl | 37218033 | 36434380 (97.89) | 28766786 (77.29) | 5fC | control |
| bham480 | 26397444 | 25795824 (97.72) | 20416563 (77.34) | 5fC | tdg |
| ear003_j1_sirna_tdg | 36652853 | 35829193 (97.75) | 28702060 (78.31) | 5fC | tdg |
| mb001_j1_hmc_r1 | 31140117 | 30420575 (97.69) | 23550574 (75.63) | 5hmC |  |
| mb002_j1_hmc_r2 | 23031379 | 22359337 (97.08) | 17378492 (75.46) | 5hmC |  |
| mb003_j1_medip_r1 | 21171139 | 19904661 (94.02) | 9617310 (45.43) | 5mC |  |
| mb004_j1_medip_r2 | 26847453 | 25896399 (96.46) | 14070566 (52.41) | 5mC |  |
| bham385 | 40026004 | 34184998 (85.41) | 26452706 (66.09) | input |  |
